# Characteristics, Outcomes and Recovery of Patients 65 Years or Older Admitted to Swedish Intensive Care Units: A Protocol for a Longitudinal Observational Multicentre Study

**DOI:** 10.1111/nicc.70109

**Published:** 2025-07-11

**Authors:** Fredrika Sundberg, Anna Kjellsdotter, Elisabeth Lindberg, Emma Backman, Åsa Israelsson‐Skogsberg

**Affiliations:** ^1^ Research, Education, Development and Innovation Department Skaraborg Hospital Skövde Skövde Sweden; ^2^ The School of Health Sciences Skövde University Skövde Sweden; ^3^ Faculty of Caring Science, Work Life and Social Welfare University of Borås Borås Sweden

**Keywords:** elderly, frailty, health, intensive care unit, older people, recovery

## Abstract

**Background:**

There are little data on the impact of frailty on critically ill older patients treated in intensive care units (ICUs) and on their characteristics and outcomes. More understanding of the longitudinal health and recovery process is needed and of the recovery traits of older patients after intensive care.

**Aim:**

This project aims to identify characteristics and outcomes in patients 65 years or older admitted to ICUs and to explore how health and recovery is experienced after discharge, with a special focus on frailty.

**Study Design:**

This research project will conduct both retrospective and prospective data collection with a sample of approximately 3200 patients. This is a longitudinal, multicentre, prospective, observational research project with a nested cohort covering 12 months of admissions and comprising four studies. The first aims to map the characteristics of patients admitted to the ICUs, their treatments and their outcomes. The second will use questionnaires to assess their health and recovery process up to 18 months after discharge. The third and fourth studies aim to describe and understand their lived experiences using research interviews, with the fourth study including only frail patients.

**Relevance to Clinical Practice:**

The project comprises studies that seek to identify the characteristics of older people admitted to ICUs, to examine how frailty impacts them and to understand what they experience during and foremost after intensive care. The project also aims to understand the facilitators and barriers to promoting health and recovery after discharge from ICUs and to contribute to the growing body of evidence supporting health and recovery initiatives. The results need to be spread and the knowledge sprung from this project may be implemented and used by intensive care unit clinicians.

**Summary**

- What is known about the topic
  ○ Approximately 50% of patients in the intensive care unit (ICU) are aged 65 years or older.
  ○ Individuals aged 65 years or older has a very high mortality risk and faces an increased risk of complications during intensive care.
  ○ Surviving patients often have difficulties recovering after discharge from ICU with substantial lingering symptoms.
- What this paper adds
  ○ Provides insights into key aspects of caring for patients aged 65 years or older.
  ○ Enhances understanding of how recovery is experienced after ICU care from the perspective of those aged 65 years or older with a special focus on frail patients.
  ○ Contributes valuable knowledge on best practices for caring for individuals aged 65 years or older in need of intensive care.

## 1 Background

The median age of patients treated in intensive care units (ICUs) is over 65 years [1], and the number of patients aged 80 years or older in ICUs has increased, now constituting one of the largest demographics in intensive care in many countries [2, 3, 4]. Older people have a higher prevalence of chronic diseases and an age‐related decrease in physiological reserves, making them more vulnerable to acute diseases [5]. Comorbidities are also associated with increased mortality in ICUs [4] and ICU patients aged 80 years or older have a one‐year mortality rate of around 60% [6, 7]. Age, functional status assessed with ADL (activities of daily living) scores, presence of an active cancer and poor nutritional status have all been identified as independent factors associated with higher 6‐month mortality in older ICU patients [8].

Physical, psychological and existential changes due to ageing contribute to an individual's vulnerability and may limit their ability to manage and cope with everyday life [4]. Patients in ICUs need holistic care from healthcare professionals who are knowledgeable about the changes associated with ageing [9]. A comprehensive assessment of older patients' needs should therefore take place at the beginning of ICU treatment and be continually reviewed [10].

Research underscores that, in this context, frailty is more important than chronological age [4]. The Clinical Frailty Scale (CFS) is a visual measure of different dimensions of frailty, which includes nine classes that integrate physiological, functional and cognitive aspects [11]. An early frailty diagnosis can improve everyday life after ICU treatment if vulnerable individuals who could benefit from follow‐up interventions can be identified [12]. Indeed, it can be challenging for older people to achieve acceptable health‐related quality of life (HRQoL) after ICU treatment [13, 14], but life can progressively become more normal, with HRQoL scores often rising over time [6, 13, 15]. The concept of recovery is then understood as an ongoing process, not a state, in which health constantly varies despite medical diagnoses [16, 17]. ICU survivors have described a sense of recovery when they first return to their own homes [18] and are again in a social context [19]. This is consistent with an earlier study that described sufficient time for recovery as an important factor [13]. Most older ICU survivors in that study needed a year after discharge from ICU to perceive their HRQoL as acceptable. But after that year they reported a willingness to be readmitted to the ICU if necessary [13].

The current project takes a caring science perspective in which the concept of health is seen in relation to each person's unique life, and, against a background of several complex diseases, health may become a process that is reliant on unique human relationships and interactions. The subjective dimension of well‐being is strongly emphasised and is understood as a search for meaning through movement between one's current reality and future possibilities, during which the perception of health and well‐being can fluctuate from minute to minute [17], and as involvement with friends and engagement in life [20].

To better understand the situation for older persons after discharge from ICU, research with a long‐term prospective design and with repeated questionnaire assessments at several time points is needed [13]. Furthermore, data on patients' time in the ICU are needed to understand their experiences, and potentially relevant factors such as age, frailty and socio‐demographic background need to be examined. Treatments given and assessments performed during their time in the ICU may also be predictive of their health, well‐being and recovery after discharge, and there remains a large gap in the literature regarding how older patients and their next of kin perceive the experience of everyday life after discharge from the ICU [13].

## 2 Aim

The overall aim is to identify characteristics and outcomes for patients aged 65 years or older admitted to ICUs and to explore how their health and recovery is experienced after discharge, with a special focus on frailty.

## 3 Methods

### 3.1 Design

This is a longitudinal, multicentre, prospective, observational study with a nested cohort of patients aged 65 years or older covering 12 months of admission at ICUs in southwestern Sweden. Data collection and analysis will be performed using various methods to study short‐ and long‐term outcomes and to explore how patients experience recovery and health after discharge. The STROBE guidelines for the conduct and dissemination of observational studies will be used [21]. The research project will comprise four studies (Table 1) with an overarching aim.

**TABLE 1:** Overview of the four planned studies.

Study	Aim	Participants	Data collection	Method of analysis
I	To investigate characteristics and outcomes of patients aged 65 years and older admitted to Swedish intensive care units (ICUs)	All patients aged 65 years and older admitted during a 12‐month nested cohort to the participating multidisciplinary ICUs will be included in the study	Data from patients' medical records and national registries	Descriptive statistics and multivariate analysis of variance
II	To study the experience of patients aged 65 years and older of health‐related quality of life (HRQoL), sleep, recovery and health at 6, 12 and 18 months after discharge from the ICU in relation to the characteristics of the care received	All patients aged 65 years and older admitted and later discharged during the nested cohort of 12 months from the included multidisciplinary ICUs will be invited to participate	Questionnaires: Rand‐36, RAIN and Pittsburgh Sleep Quality Index (PSQI) administered 6, 12 and 18 months after discharge from the ICU	Descriptive statistics and multivariate analysis of variance
III	To illuminate the meanings of health and recovery among patients aged 65 years and older after intensive care	10–15 non‐frail patients aged 65 years and older scoring 1–3 on the Clinical Frailty Scale (CFS)	Individual interviews 12–18 months after discharge from the ICU	Phenomenological or hermeneutical analysis
IV	To illuminate the meanings of health and recovery among frail patients aged 65 years and older after intensive care	10–15 frail patients aged 65 years and older scoring 4–9 on the CFS	Individual interviews 12–18 months after discharge from the ICU	Phenomenological or hermeneutical analysis

To address the objective in Study I, the following research question has been formulated:

– Are there disparities in the care interventions and medical treatments provided based on frailty among older patients admitted to the ICU?

To address the objective in Study II, the following research questions have been formulated:

– Is frailty associated with HRQoL, recovery and health among older patients admitted to the ICU?
– Are specific treatments in intensive care associated with sleep quality, HRQoL and recovery after discharge from the ICU?

#### 3.1.1 Setting

The research project will involve six multidisciplinary ICUs in southwestern Sweden, a region home to 1.77 million of Sweden's 10.6 million inhabitants. This area is representative of the rest of Sweden, adhering to the same legislations and national guidelines in healthcare. Sweden has among the lowest ratio of intensive care beds in Europe, with five beds per 100 000 people (accessibility index [AI]) [22]. The setting for this project includes both urban and rural areas with both university hospitals and county hospitals. Notably, the university hospital in this region is the largest in Sweden. In 2024, 39 936 patients were admitted to Swedish ICUs, with 5382 of these patients in this southwestern region.

#### 3.1.2 Study Population

All patients aged 65 years or older admitted within the 12‐month nested cohort to the multidisciplinary ICUs will be included in the research project. In 2023, the included ICUs had 5987 admissions, of whom 3163 were patients aged 65 years or older and 946 were 80 years or older. We estimate similar admissions to this research project, but interim analysis after 12 months of data collection is planned [23].

#### 3.1.3 Outcome Measures

The short‐ and long‐term outcomes for Study I include hospital mortality, post‐discharge mortality, length of stay (LoS) in the ICU and in hospital. Additional factors may be considered based on the data received in relation to variables (Box 1). For instance, factors such as the incidence of ICU‐acquired infections, the duration of mechanical ventilation, the frequency of readmissions and the need for rehabilitation post‐discharge could provide valuable insights. These outcomes may be included if the collected data support such analyses and if they align with the study's objectives. This approach ensures flexibility while adhering to the study's protocol and maintaining its scientific rigour.

The outcomes for Study II are those for Study I plus self‐reported health, quality of life, sleep and overall recovery in relation to frailty (measured using the CFS on a scale of 1–9, with 4 or more regarded as frail). The qualitative studies (Studies III and IV) will focus on lived experiences of health and the recovery process after being discharged from the ICU.

BOX 1 **Variables to be included in the research project.**

**Table:** 

Patients' characteristics	– Age – Sex – Height – Weight – Socio‐demographic background – Preadmission comorbidities (hypertension, coronary artery disease, chronic heart failure, chronic obstructive pulmonary disease (COPD), diabetes mellitus, chronic kidney disease, cerebral infarction etcetera)
Treatments in the ICUs	– Continuous renal replacement therapy (CRRT) – High‐flow nasal cannula (HFNC) – Invasive and non‐invasive ventilation – Vasopressors/inotropy – Limitations of life sustaining therapies (LST) – Sedation
Events and consequences of critical illness and treatment in the ICU	– Adverse events – Delirium – Acuity scoring system – Pain

### 3.2 Data Collection

This research project will be based on quantitative measures and qualitative data from the patients' medical records, national registries, questionnaires and individual interviews.

#### 3.2.1 Patients' Medical Records and National Registries

Data regarding patient status at admission, medical and caring activities during the ICU stay and outcome (status at discharge) will be collected from the patients' medical records at the respective hospitals. Additional information, such as socio‐demographic background, will be collected through Swedish national quality registries, the Swedish Intensive Care Registry (SIR), the National Board of Social Affairs and Health and Swedish governmental registries (SCB).

#### 3.2.2 Questionnaires

In addition to socio‐demographic data about the participants, data will be obtained using validated self‐report questionnaires that assess HRQoL, recovery following intensive care and sleep quality over the previous month (Table 2). These data will be gathered at 6‐, 12‐ and 18‐months following discharge from the ICU.

**TABLE 2:** Questionnaires used in the study.

Questionnaires	No. of items, score range and constructs	Measures
The RAND 36‐Item Short Form Health Survey [24]	36 items, 0–100 range for each dimension, with higher scores indicating better health‐related quality of life	Assesses physical, emotional and social functioning in daily life over the previous 4 weeks in eight domains: physical functioning (PF), role limitations due to physical health (RP), bodily pain (BP), general health (GH), vitality (VT), social functioning (SF), mental health (MH) and role limitations due to emotional health (RE)
The Recovery After Intensive Care (RAIN) instrument [25]	20 items (measured on a 5‐point Likert scale), 20–100 range, with higher scores indicating better recovery	Assesses recovery following intensive care using six subscales: (1) looking forward, (2) supportive relationships, (3) existential ruminations, (4) re‐evaluation of life, (5) physical and mental strength and (6) need for social support
The Pittsburgh Sleep Quality Index (PSQI) [26]	19 items, 0–21 range for seven component scores, with higher scores indicating worse sleep and scores over 5 interpreted as poor sleep quality	Assesses seven dimensions of sleep over the past month: subjective sleep quality, sleep latency, sleep duration, habitual sleep efficiency, sleep disturbances, use of sleeping medication and daytime dysfunction

In addition to collecting socio‐demographic data about the participants, validated self‐report questionnaires will be used to gather information on various aspects of health and recovery (Table 2). The RAND 36‐Item Short Form Health Survey (The Rand‐36) [24] will assess physical, mental and social dimensions of health‐related quality of life (HRQoL), the Recovery After Intensive Care (RAIN) instrument [25] will evaluate recovery following intensive care, and the Pittsburgh Sleep Quality Index (PSQI) [26] will measure sleep and sleep quality over the past month. Data collection will occur at 6, 12 and 18 months after ICU discharge.

#### 3.2.3 Qualitative Interviews

This part of the research project (Studies III and IV) will be situated within a phenomenological framework [24], as we attempt to understand and describe what the experience means for the person who has lived it—in this case, the lived experience of health and recovery after intensive care. A phenomenological framework requires researchers to examine the issue from the perspective of those who have first‐hand experience [24].

Data collection will use individual interviews characterised by openness, with the aim of creating a deeper understanding of the investigated phenomena: how HRQoL, recovery and health are experienced after intensive care [25]. The number of individual interviews will be 10–20 per study, with the content of the interviews being the determining factor in the final number conducted. Therefore, frequencies will not be of interest; rather, the focus will be on the phenomena themselves. The aim is to enable participants to describe variations of the phenomenon. Consequently, the goal is to recruit participants who represent a diverse range of gender, age and reasons for their intensive care stay. The individual interviews will be conducted at 12–18 months following discharge from the ICU (see Table 1). Interviews of non‐Swedish speakers will take place with an interpreter. The interviews will take place in undisturbed locations chosen by the participants—preferably their homes—and be audio‐recorded and transcribed verbatim.

Three months after discharge from the ICU, the patients selected for the study will be contacted and invited to participate. Patients who agree will then be provided more information by a designated person in the research group and scheduled for an interview.

### 3.3 Ethical Considerations

The international guidelines for scientific research, as stated in the Declaration of Helsinki, will be considered regarding autonomy, integrity, beneficence non‐maleficence and justice (2013). The study has been approved by the National Ethical Review Board on 29 May 2024 (No. 2024‐01205‐01). All managers of the included ICUs have approved access and participation in the research project. Informed and written or verbal consent will be obtained from the participants in the study. They will also be informed that they can end their participation in the study at any time without explanation or consequence. No side‐effects are expected. Study findings will be presented at national and international conferences and published in peer‐reviewed journals.

## 4 Analyses and Data Processing

Data will be analysed using both quantitative and qualitative methods. In Study I, all patients admitted to the ICUs will be included, so neither sample size nor power calculations have been performed. Descriptive statistics—frequencies and percentages for categorical variables and medians with quartiles, means and/or standard deviations (SDs) for continuous variables—will be used to provide general descriptions of the variables. The relationships between variables will be analysed using correlation analyses, and multivariate models will be constructed appropriate to the data and the outcome variable of interest. For instance, HRQoL and LoS are continuous variables, which may be analysed using variance analysis models, while mortality, need for dialysis and ventilator use may be analysed using logistic regression when treated as binary outcomes or using Kaplan–Meier survival analysis when measured as time to event. To consider possible confounders, suitable multivariable models, such as logistic regression or Cox regression, will be used. All statistical analyses will be performed using IBM SPSS (version 28; IBM, Armonk, New York, USA), with a significance level of 5% and a 95% CI.

In the qualitative studies (Studies III and IV), both interpretative hermeneutic and descriptive phenomenological approach based on reflective lifeworld research (RLR) will be used [24]. Each interview will be transcribed verbatim, and all collected data will be seen as a single piece of text. In the first analysis phase, the text will be read in an open manner to gain familiarity with the data. The next phase seeks meanings of lived experiences, with the help of the researcher making marks in the text and writing down own words and notes. When meanings have emerged, the researcher can see similarities and differences in the informants' stories. These are brought together to create a new whole. Phenomenological methodology can be described as a movement from whole to part, which then forms a new whole. For the phenomenon to be given the opportunity to show itself in all its dimensions, the entire research process must be characterised by openness and responsiveness [24].

The quality and richness of the data will guide the subsequent steps. The phenomena will comprise nuances and constituents and will be illustrated with interview quotes, which will also support credibility as a part of the trustworthiness of the analysis [27].

## 5 Discussion

This project uses both quantitative and qualitative methodologies. The quantitative part will assess patients aged 65 or older admitted to the ICU in terms of their numbers, reasons for admission, frailty, treatments and outcomes. This will be elaborated upon using self‐reported experiences of HRQoL, recovery and sleep after discharge from the ICU, with an 18‐month longitudinal follow‐up.

The qualitative aspect will contribute knowledge about how health and recovery are experienced after intensive care by those over 65. This group is heterogeneous, with different diagnoses and treatment strategies, and previous studies have remarked upon the lack of knowledge of their experiences of health and recovery [13].

Inclusion criteria for research studies often require informants be able to speak and understand the relevant language, sustain cognitive health and provide their consent in written form [28]. Older people who have survived critical illness and spent time in the ICU may have difficulties in providing informed consent due to impaired cognitive health and frailty, which can result in their exclusion from a study or to not being asked to participate in the first place. Overly rigid inclusion criteria mean that we have less knowledge about older people with greater care requirements and about their wishes and needs. We cannot address all these issues in our upcoming research; however, we will attempt to address some of them, such as using a translator if needed. Since intensive care nurses are responsible for data collection and participant inclusion, there may be participants included who would otherwise have been excluded due to fear or lack of experience in interacting with these individuals. Although we will always be guided by our ethical and moral compass, we will ensure that informed consent is both truly informed and understood. In a systematic review, Chang et al. [29] found that approximately 95% of the studies they reviewed had evidence of ageism, with exclusion of older people common even in areas in which they were the most frequent recipients of care.

Research also tends to exclude people who cannot themselves give traditional consent to participation [28]. In the present study, we want to overcome this exclusion of older people and obtain a broad perspective that includes the valuable insights that come from lived experiences. It is therefore important that we offer to include those who can and want to participate. Ethical reflections in relation to fragility are critical, with prolonged engagement, asking for consent on several occasions and repeated, but shorter, interviews [30] being examples of valuable strategies to support older participants, as well as conducting interviews with support of an interpreter to include non‐Swedish speaking persons. One reason for excluding groups from research is their perception that data collection is exhausting, but research has shown that participating in an interview, with the opportunity to reflect on questions of importance to one's existence, can create a sense of well‐being and generate new insights [31]. For the oldest, the opportunity to share their stories and to be listened to is particularly important for well‐being [32].

## 6 Strengths and Limitations

The present study addresses a timely concern because caring for older patients is common in the ICU yet research on patients aged 65 years or older in need of intensive care is inconsistent and diverse; more knowledge is therefore needed about best practices in care and follow‐up at both the individual and group levels. The results of the current study may not be generalisable due to the heterogeneity of the participants, which could be regarded as a weakness; however, the qualitative part of the project has a phenomenological design with a caring science perspective, which considers the lifeworld of older patients as unique and treats human existence as a whole, regardless of diagnosis. This perspective is regarded as a strength and offers valuable knowledge about how health and recovery can be experienced by older people after leaving the ICU.

There are some barriers and limitations to conducting this research project that will need to be addressed. The first is the geographic area, throughout which the researchers will need to travel to reach the different hospitals and for interviews in participants' homes, if requested. This is a challenge of which we are aware, and the project has been designed to be carried out over several years to fully address it. A second limitation could be heterogeneity among the patients, which may affect generalisability of the results. However, even if the participants in Studies III and IV are limited and heterogenous, the results can, due to the phenomenological approach, be transferred to other contexts by presenting the results as a structure of meanings in which the essential structures and nuances of the phenomena are presented [33]. There is also the concern that there will be too few participants who are both older and frail, therefore may not be able to participate due to circumstances. However, given that there are 3200 eligible patients from the start, we do not anticipate a sample size that is too small. If necessary, we will extend the data collection period. Given the comprehensive nature of the study designs, the focus on older individuals, and the meticulous and detailed descriptions of the studies and their context, the knowledge generated from the project as a whole may be generalisable and transferable to other contexts.

## 7 Implications of the Research

In conclusion, this project offers an important contribution to increase knowledge of care and treatment of critically ill patients aged 65 years or older. It will illuminate the vulnerable transition from critical illness to a life that may differ from pre‐ICU admission, detailing the clinical course and the factors influencing this process. Despite its limitations, we believe the findings of this study will provide a firm foundation for future research.

## Ethics Statement

International guidelines for scientific research, as stated in the Declaration of Helsinki, will be considered regarding autonomy, integrity, beneficence, non‐maleficence and justice [32]. The study received approval from the National Ethical Review Board on 29 May 2024 (No. 2024‐01205‐01). Informed and either written or verbal consent will be obtained from the participants, who will be notified that they can end their participation in the study at any time without explanation or consequence. No side effects are expected. Study findings will be presented at national and international conferences and published in peer‐reviewed journals.

## Consent

The authors have nothing to report.

## Conflicts of Interest

The authors declare no conflicts of interest.

## Data Availability

Data sharing is not applicable to this article as no new data were created or analyzed in this study.
